# Anatomical variants of renal veins: A meta-analysis of prevalence

**DOI:** 10.1038/s41598-019-47280-8

**Published:** 2019-07-25

**Authors:** Sorin Hostiuc, Mugurel Constantin Rusu, Ionut Negoi, Bogdan Dorobanțu, Mihai Grigoriu

**Affiliations:** 1 Carol Davila University of Medicine and Pharmacy, Faculty of Dental Medicine, Department of Legal Medicine and Bioethics, Bucharest, Romania; 2 Carol Davila University of Medicine and Pharmacy, Faculty of Dental Medicine, Department of Anatomy, Bucharest, Romania; 3 Carol Davila University of Medicine and Pharmacy, Faculty of Medicine, Department of Surgery, Bucharest, Romania; 4 Clinical Emergency Hospital, Bucharest, Romania; 5 Carol Davila University of Medicine and Pharmacy, Faculty of Medicine, Department of Surgery, Bucharest, Romania; 6 Fundeni Clinical Institute, Bucharest, Romania; 7 Carol Davila University of Medicine and Pharmacy, Faculty of Medicine, Department of Surgery, Bucharest, Romania; 8 University Emergency Hospital Bucharest, First Surgery Clinic, Bucharest, Romania

**Keywords:** Kidney, Kidney

## Abstract

The main aim of this article is to establish the actual prevalence of renal vein variations (circumaortic renal vein, retroaortic renal vein, double renal vein), and to increase awareness about them. To this purpose, we have performed a meta-analysis of prevalence, using the MetaXL package, We included 105 articles in the final analysis of prevalence, of which 88 contained data about retroaortic renal vein, 84 – about circumaortic renal vein, and 51 - about multiple renal veins. The overall prevalence for retroaortic renal vein was 3% (CI:2.4–3.6%), for circumaortic renal vein − 3.5% (CI:2.8–4.4%), and for multiple renal veins - 16.7% (14.3–19.2%), much higher on the right 16.6 (14.2–19.1%) than on the left side 2.1 (1.3–3.2%). The results were relatively homogenous between studies, with only a minor publication bias overall.

## Introduction

The anatomy of the renal veins was studied by many authors, due to its major implications in abdominal surgery (e.g. nephrectomy, in kidney transplantation). Knowledge regarding the morphology and prevalence of vascular abnormalities is also of an uttermost importance in laparoscopic surgery when entering the paraaortic region, as the repair of renal vessels is much more difficult compared to open surgery, often causing hemorrhage, a need for transfusion, or conversion to laparotomy^1^. Various anatomical variants of the renal veins were associated with varicocele^2^, nutcracker syndrome^3^, pelvic congestion syndrome^3^, hematuria, low-back pain^4^, or renal ectopy^5^. There are three main types of anatomical variants of renal veins: multiple renal veins, in which are identifiable two or more renal veins, either uni or bilaterally; retroaortic left renal vein (RLRV), in which the renal vein has a retroaortic course before entering the inferior vena cava; and circumaortic left renal vein (CLRV), in which there are two or more renal veins forming a ring around the aorta. The anatomy and surgery manuals often overlook these anatomical variants, increasing the risk for less experienced surgeons to damage them during surgery. The prevalence of the main anatomical variants of the renal vessels is variable in the scientific literature. For the RLRV the prevalence varies in different studies between under 1%^6^, and close to 10%^7,8^. For the CLRV, the quoted prevalence ranges from below 1%^3,9,10^ to over 15%^11^. Multiple renal veins (MRVs), have a prevalence ranging from 2%^12^ to over 40%^13^. The main aim of this article is to establish the actual prevalence of these anatomical variants (RLRV, CLRV, MRVs).

## Materials and Methods

We performed the study according to the PRISMA guidelines for reporting systematic reviews and meta-analyzes of observational studies in epidemiology^14^.

### Selection criteria

Inclusion criteria: studies that contained data from which could estimate the prevalence of the main renal vein variations: retroaortic left renal vein, circumaortic renal vein (renal vein collar), multiple renal veins, on various population groups. We used as exclusion criteria: (1) no relevant information to reconstruct the data needed for analysis; (2) studies made on less than 20 subjects; (3) case series/case reports. For articles not found in online databases, but for which we could obtain numerical data from secondary sources, we used the secondary source-based information.

### Search method

We analyzed the results from three databases: Web of Science, Scopus, and Pubmed, by using the following keywords: “renal collar”, “Circumaortic renal vein”, “double renal vein” “retroaortic renal vein”, with a timeframe that ranged from the beginning of each database to May 2018. We preferred not to use additional, restrictive criteria (e.g. article type) as other assortments (letters, case presentations, reviews) might have added relevant data to the meta-analysis (discussions, finding other appropriate articles). The reference list of each relevant one was scrutinized for other relevant studies to be included in the meta-analysis. We imported the references, abstract and full text (if available) into the Mendeley Desktop software.

### Data collection and analysis

For each study, two reviewers, working independently, performed the database research, extracted the data and included it in Excel Datasheets. If discrepancies were found, the articles into question were reviewed by a third reviewer. We summarized the following information: study, name of the authors, year, total number of cases, country, the general inclusion and exclusion criteria, the number of cases with various renal vein variations, including subtypes for RLRV, the gender for RLRV and CLRV, the detection method, the risk of bias, and the quality score. If the data was obtained from secondary sources, we only used the data that was available in these secondary sources and the risk of bias and the quality score were not computed.

### Risk of bias

Two reviewers assessed separately the risk of bias qualitatively, based on a methods we have previously used in another meta-analyses of prevalence^15^. When the opinions of the two reviewers diverged, regarding the risk of bias of a specific study, a third reviewer reassessed the article, and decided the final risk of bias, used in our analysis. We analyzed selection bias (the presence of inclusion and exclusion criteria, type of study), multiple publication bias, measurement bias (method used, with autopsy and high-resolution CT imaging being considered having a lower bias compared to venography), statistical reporting bias (statistical analysis performed with the data, complete description of the data). Based on these elements, we separated the studies in three subgroups: high risk of bias, moderate risk of bias and low risk of bias. A high risk of bias was considered when the inclusion and exclusion criteria were undefined/improperly defined, authors have published more than one article on similar populations, the reviewers being unable to properly assess the clear separation of the study groups in different articles, the use of venography or low-resolution CT, the study was not performed specifically to assess the presence of venous structures, the data was very scarcely presented, the number of cases was low. A low risk of bias was assessed when the inclusion and exclusion criteria were properly defined, the variants were detected through high-resolution CT, anatomy or surgery, during studies aimed specifically for the detection of venous variants, the number of subjects was high. A moderate risk was assessed in studies with intermediate characteristics.

### Quality assessment

We performed the quality assessment using four scales from the Quality in Prognostic Studies Tool^16^ (participants, outcome measurement, confounding, statistical analysis and reporting). For each remained subscale (study participation, prognostic factor measurement, outcome measurement, study confounding, statistical analysis and reporting), we graded each study as low quality (0 points), intermediate quality (1 point) or high quality (2 points). This method was previously used by the authors in meta-analyses of prevalence^16^.

### Statistical analysis

We determined the effect size using a random effects model computed in Microsoft Excel 2016 with the MetaXL add-on version 5.3. For each group and subgroup, we performed a forest plot. For the analysis of publication bias, we used the funnel plot and the LFK index. For the prevalence analysis we performed the double arcsin prevalence transformation, we used a continuity correction of 0.5 and 95% confidence intervals. Forest plots were done using Microsoft Excel 2016 with the MetaXL add-on 5.3. The actual prevalence can be obtained by multiplying with 100 the results from the meta-analysis of prevalence.

## Results

### Search synthesis

**Figure 1:**
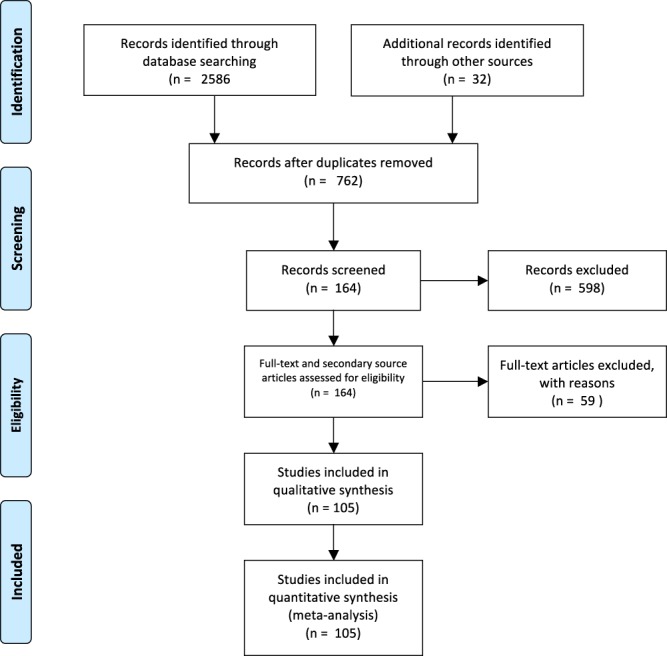
Search synthesis. PRISMA flow diagram. The PRISMA Statement and the PRISMA Explanation and Elaboration document are distributed under the terms of the Creative Commons Attribution License, which permits unrestricted use, distribution, and reproduction in any medium, provided the original author and source are credited.

During the initial database research, we obtained 2586 (Table 1) articles from which, after deleting duplicates and irrelevant studies we selected 132 to be further scrutinized (128 by the first reviewer, 123 by the second, 119 being common). By analyzing their references, we found another 32 potentially relevant articles that were also downloaded (30 and 32 articles, by reviewer 1 and 2, respectively). From the 164 articles, 105 were included in the final analysis of prevalence, of which 88 contained data about RLRV, 84 – about CLRV, and 51 about multiple RVs. Details about the search synthesis are presented in Fig. 1. We detailed the papers contained in the meta-analysis in Table 2.

**Table 1:** Keyword search.

Keyword search	Pubmed	Scopus	Web of Knowledge	Number of articles
Retroaortic renal vein	188	227	137	552
Renal venous collar	37	34	22	93
Circumaortic renal vein	113	137	83	333
Double renal vein	493	776	339	1608
Total	831	1174	581	2586

**Table 2:** Studies included in the analysis.

Study	Country	Type/Inclusion criteria	Exclusion criteria	Method	No cases
Alexander, 1981^31^	US	Retrospective		CT	1200
Aljabri, 2001^32^	Canada	Retrospective, randomized	Technical reasons	CT	1788
Anson, 1947^33^	US	Various		Autopsy	425
*Anson, 1961* (*Satyapan, 1999*)^19^	US			Autopsy	100
Apisarnthanarak, 2012^21^	Thailand	Living related kidney donors, consecutive		CT	65
Arslan, 2005^2^	Turkey	Consecutive		CT	1125
Atalar, 2012^3^	Turkey	Retrospective	LRV could not be evaluated	CT	739
Ayaz, 2016^34^	Turkey	Consecutive patients who underwent routine oncological PECT/CT examinations		CT/PET	222
Ballesteros, 2014^18^	Colombia	Various, metisho subjects		Autopsy	156
Baptista, 1997^35^	Brasil	Living donor nephrectomies		Surgery	342
Beckmann, 1980^36^	US	Consecutive		Venography	132
Benedetti-Panici, 1994^37^	Italy	Patients with various oncological disorders, operated with systematic aortic and pelvic lymphadenoectomy		Surgery	309
Bouali, 2012^38^	France	Various	Abdominal aortic prosthesis, aneurysm, history of kidney surgery, kidney atrophy, poor quality of the examination or enhancement	CT	120
Boyaci, 2014^39^	Turkey	Patients with abdominal problems		CT	746
Clnar, 2016^40^	Turkey	Various reasons for referral for an imaging of the abdominal aorta and its branches	Previous abdominal aortic surgery, failure to assess renal vascular anatomy	CT	504
Costa, 2011^41^	Brasil	Patients undergoing nephrourecterectomy		Surgery	254
*Davis, 1958 (Satyapal, 1999)* ^42^	US			Autopsy	100
Davis, 1968^43^	US			Autopsy	270
Dilli, 2012^44^	Turkey	Retrospective, patients undergoing lumbar imaging for neurological disorders		MRI	2644
Dilli, 2013^45^	Turkey	Retrospective, various abdominal problems		CT	1204
Duques, 2002^23^	Brasil	Various, metisho subjects		Autopsy	34
Duran, 2016^8^	Colombia	Various		Autopsy	23
Eisendrath, 1920^13^	US			Autopsy	218
Ellis, 1986^46^	US	Nonspecific		CT/MRI	241
*Făgărășanu, 1938 (Satyapal,1999; Yi*,2012)^47^					71
*Froriep, 1895 (Satyapal, 1999)* ^48^					28
*Gerard, 1921 (Satyapal,1999; Yi,2012)* ^49^					225
Gillaspie, 1916^50^	US	Various		Autopsy	33
Gillot, 1978^25^	France			Autopsy	322
Gupta, *2012*^29^	India	Various		Autopsy	30
Hassan, 2017^51^	Egypt	Various		Autopsy	63
Heidler, 2015^52^	Austria	Patients with suspected stone disease or neoplasms		CT	7929
Hicks, 1995^53^	US	Prospective, patients referred for IVC filter placemembt or cavography	Abnormal serum creatinine, emergent procedure, internal jugular vein access, occlusion of the IVC, allergy to intravenously administered contrast material, procedure performed outside the interventional radiology department	Venography/Cavography	108
Hoeltl, 1990^10^	Austria	Unselected patients		ct	4520
Hoeltl, 1990^10^	Austria	Patients undergoing surgery for major retroperitoneal operations for urological disorders.		surgery	215
Hoeltl, 1990^10^	Austria	Unselected patients		autopsy	354
Holden, 2005^54^	New Zeeland	Renal donors		ct	100
Holt, 2007^55^	UK	Patients with testicular germ cell tumors		surgery	278
*Hovelacque, 1914 (Satyapal 1999)* ^6^					20
*Izumiyama, 1997(Satyapal 1999)* ^6^	Japan			Autopsy	266
*Jambreau, 1910 (Satyapal 1999)* ^6^ *)*	France			Autopsy	24
Janschek, 2004^17^	Austria	Unselected white cadavers		Autopsy	119
Karaman, 2007^9^	Turkey	Patients with urological or non-urological symptoms		CT	1856
Karazincir, 2007^56^	Turkey	Patients with varicocele versus a control group		Color Doppler ultrasonography	277
Kaufman, 1995^30^	US	Patients with abdominal aortic aneurysm, aortoiliac occlusive disease, renal artery stenosis		MRI	150
Kawamoto, 2005^57^	US	Potential laparoscopic living renal donors		CT	100
Klemm, 2005^1^	Germany	Patients undergoing laparoscopic infrarenal paraaortic lymphadenectomy for various oncological disorders		Surgery	86
Koc, 2007^58^	Turkey	Consecutive adult patients	Poor opacification, previous surgery, large abdominal mass	CT	1120
Kramer, 1978^59^	South Africa	Various		Autopsy	193
Kulkarni, 2011^60^	US	Potential kidney donors		CT/Surgery	102
Kumaresan, 2016^61^	India	Living kidney donors		CT	100
Lien, 1977^62^	Norway	Patients with suggested or confirmed testicular tumors	Associated pathological changes	Phlebography	100
Lin, 2004^63^	US	Living kidney donors		Laparoscopy	170
Martinez-Almagro, 1992^64^	Spain	Various		CT, Surgery	218
Martinez-Almagro, 1992^64^	Spain	Various	Vascular pathology or previous retroperitoneal surgery	Autopsy	116
Mayo, 1983^65^	Canada	Various		CT	1140
*Merklin, 1958 (Satyapal, 1995)* ^66^					185
Monkhouse, 1986^67^	UK	White European		Autopsy	57
Mosnier, 1978^68^	France	Various		Autopsy	20
Namasivayam, 2006^69^	US	Kidney donors		CT	48
Namburu, 2017^70^	India	Various		Autopsy	60
*Natsis, 2008* ^71^	Greece			CT	319
Nishimura, 1986^72^	Japan	31 patients with renal hematuria of unknown origin and 9 controls		Venography	40
Okamoto, 1990^73^	Japan	Various		Autopsy	270
Ortmann, 1968^74^	Germany	Various		Autopsy	79
Pandya, 2016^75^	India	Potential kidney donors		CT	200
Pick, 1940^11^	US	Various		Autopsy	200
Pollack, 1986^76^	Germany	Various, for transplantation		Autopsy	400
Poyraz, 2013^77^	Turkey	Consecutive	Various congenital and acquired kidney diseases	CT	1000
Pozniak, 1998^78^	US	Potential renal transplant donors		CT	205
Raman, 2007^79^	US	Potential kidney donors		CT	126
Rashid, 2014^80^	Iran	Potential living kidney donors		CT	100
*Reed, 1982 (Atalar, 2012)* ^3, 81^				CT	433
*Reginelli, 2015* ^82^	Italy	Various		CT	921
Reis, 1959^83^	US	Various		Autopsy	500
Resorlu, 2015^84^	Turkey	Various	Pathologies causing haematuria or patients with urological congenital disorders	CT	680
*Ross, 1961* ^85^		Various		Autopsy + Aortograms	34
Royster, 1974^86^	US			Autopsy	159
Royster, 1974^86^	US	Surgery for abdominal aortic aneurysm or aortoiliac occlusive disease		Surgery	228
Rydberg, 2001^87^	US	Living kidney donors		Surgery	52
Sahani, 2005^88^	US	Living kidney donors		CT	94
Sasaki, 2000^89^	US	Living renal donor-recipient pairs		Surgery	100
Satyapal, 1999^6^	South Africa	Various		Autopsy/venogram/surgery	1008
Satyapal,1995^66^	South Africa	Various	Abdominal trauma, previous surgical exploration of the abdomen, abnormal intra-abdominal macroscopic pathology	Autopsy	153
Schmidt, 1975^7^	Germany	Various		Autopsy	231
Seib, 1934^90^	US	Various. For RAA, we included both renoaortic renal vein, and renocaval arch		Autopsy	230
Shaheem, 2018^12^	Pakistan	Various, with well-preserved renal vessels	Diseased kidneys, injuries to renal veins and inferior vena cava	Autopsy	50
Shindo, 2000^91^	Japan	Surgery for aneurysmal disease or arterial occlusive disease			166
*Soloweitschick, 1899* ^92^	Germany			Autopsy	130
Sosnik, 2017^93^	Poland	Various		Autopsy	550
*Srinivasan, 1979 (Yi, 2012)* ^94, 95^				Autopsy	120
Staśkiewicz, 2016^96^	Poland	Various	﻿Insufficient contrast enhancement of renal vessels, single, transplanted or horseshoe kidneys	CT	996
Șahin, 2014^97^	Turkey	Various	Poor diagnostic quality, nephrectomy	CT/MRI	2189
Tao, 2013^98^	China	Various	Technique related, congenital diseases of the kidney, renal tumors	CT	378
Tombul, 2008^99^	Turkey	Living kidney donors		CT	60
Trigaux, 1998^100^	Belgium	Consecutive		CT	1014
Turkvatan, 2009tur^101^	Turkey	Living kidney donors		CT	59
*Weinstein, 1940 (Satyapal, 1995)* ^24, 102^		Assessment for kidney transplantation		Autopsy	203
Yagci, 2008^103^	Turkey	Consecutive		CT	783
Yeh, 2004^104^	US	Patients with hematuria or suspected aortic dissection in the retrospective group + a prospective group		CT	186
*Yeşildağ, 2004 (Atalar, 2012)* ^3, 105^	Turkey			CT	1003
Yoshinaga, 2000^106^	Japan	Various		Autopsy	203
Zamboni, 2010^107^	US	Living kidney donors and patients	﻿Less than 18 years old, situs inversus viscerum, severe artefacts impairing accurate evaluation, congenital diseases of the kidneys and renal tumors	CT	54
Zhu, 2015^108^	China	Various		CT	1452
Zumstein, 1896^109^	Germany	Various		Autopsy	220

### Quality and risk of bias

Based on the inclusion criteria, we obtained a total number of 105 studies, of which of a high quality (between 6 and 8 points) were considered 28 articles, of a medium quality (between 3 and 5 points) – 39 articles, of a low quality (between 0 and 2 points) – 21 articles, and for 17 we could not obtain a full electronic text of the manuscript, and therefore the quality score could not be computed. A low bias was assessed in 19 articles, a moderate bias in 53, and a high bias in 17. The number of studies included for each sub-analysis is presented in the respective subheading.

### Retroaortic left renal vein

**Figure 2:**
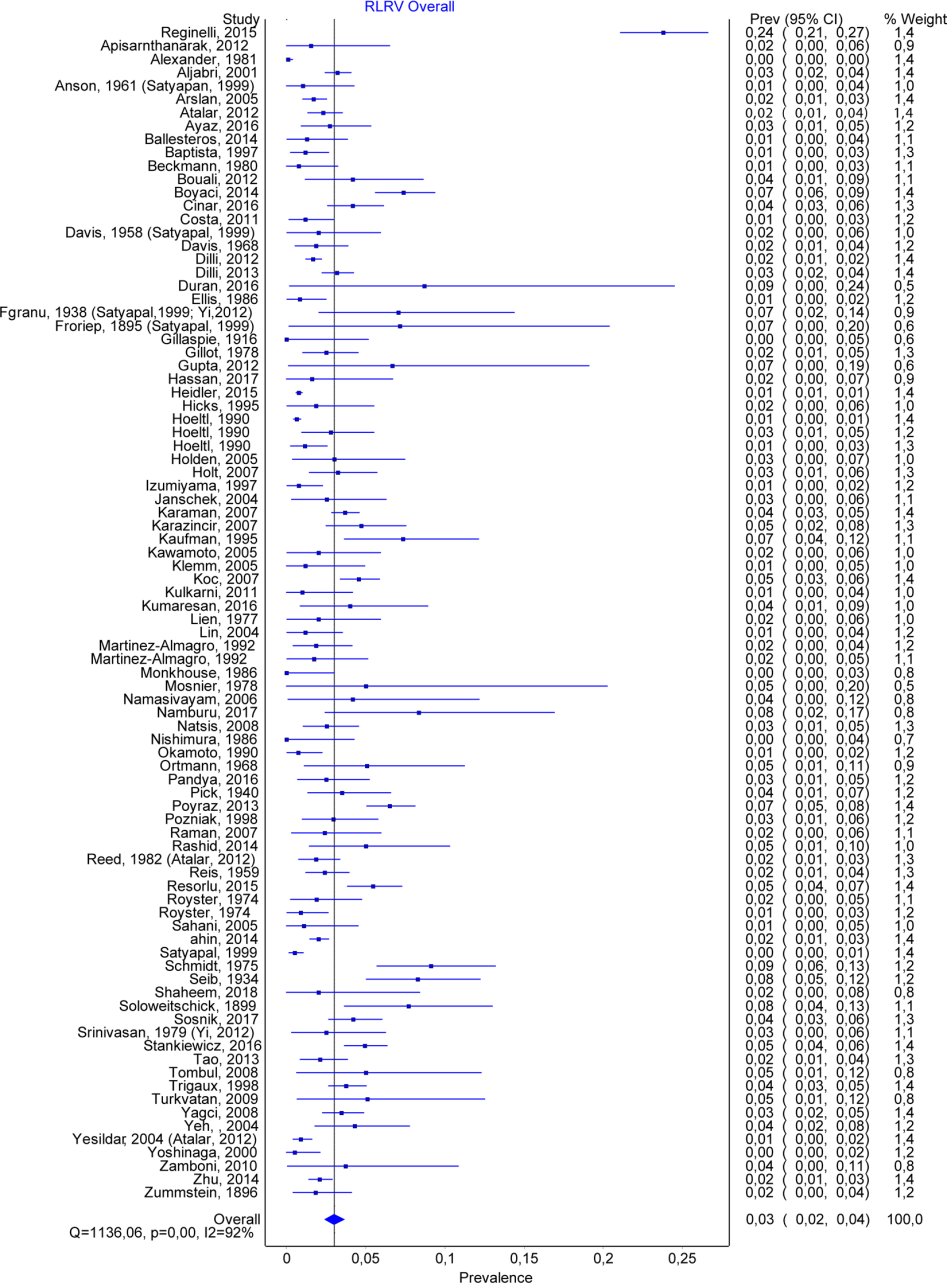
RLRV. Overall prevalence.

**Figure 3:**
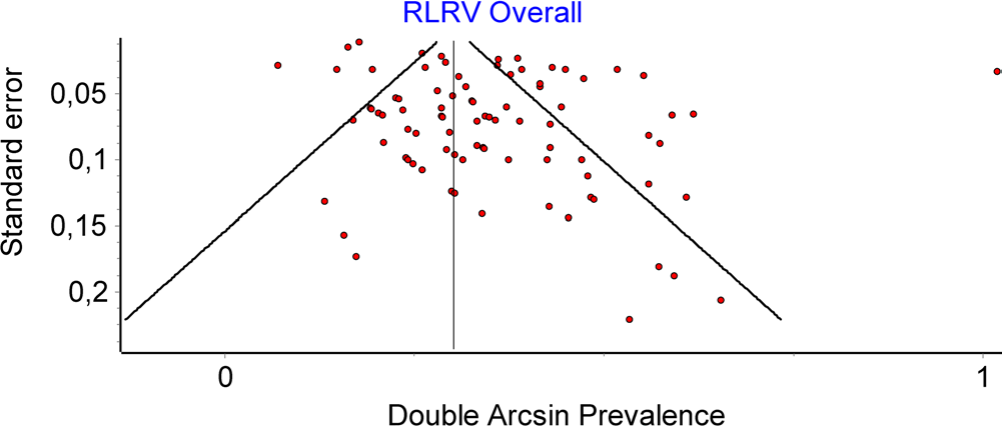
RLRV. Funnel plot.

A total number of 88 studies allowed us to estimate the prevalence of RLRV, containing 47461 subjects, of which 1287 were positive. The overall prevalence for RLRV was 0.030 (CI:0.024–0.036) (Fig. 2). The publication bias was minor, with an LFK index of 1.87. See also Fig. 3 (funnel plot). By comparing the prevalence depending on the method, we found very similar results, with a prevalence of 0.031 (0.022–0.041) for autopsy, 0.035 (0.024–0.046) for CT, and 0.02 (0.013–0.28) for surgery. Nineteen studies separated the cases based on gender. For men, the overall prevalence was 0.036 (0.026–0.048), while for women – 0.031 (0.019–0.046).

### Circumaortic renal vein

**Figure 4:**
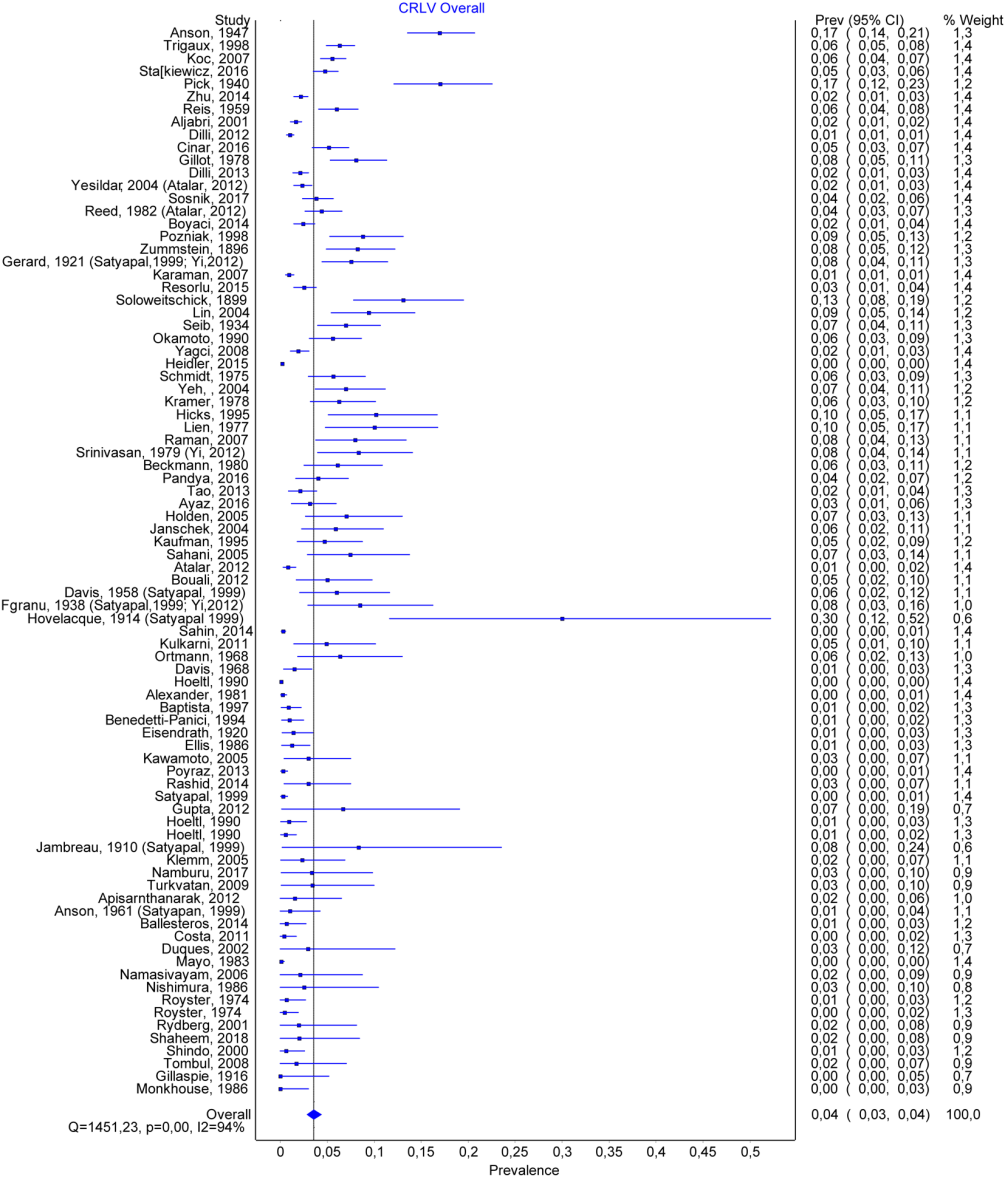
CLRV. Overall prevalence.

**Figure 5:**
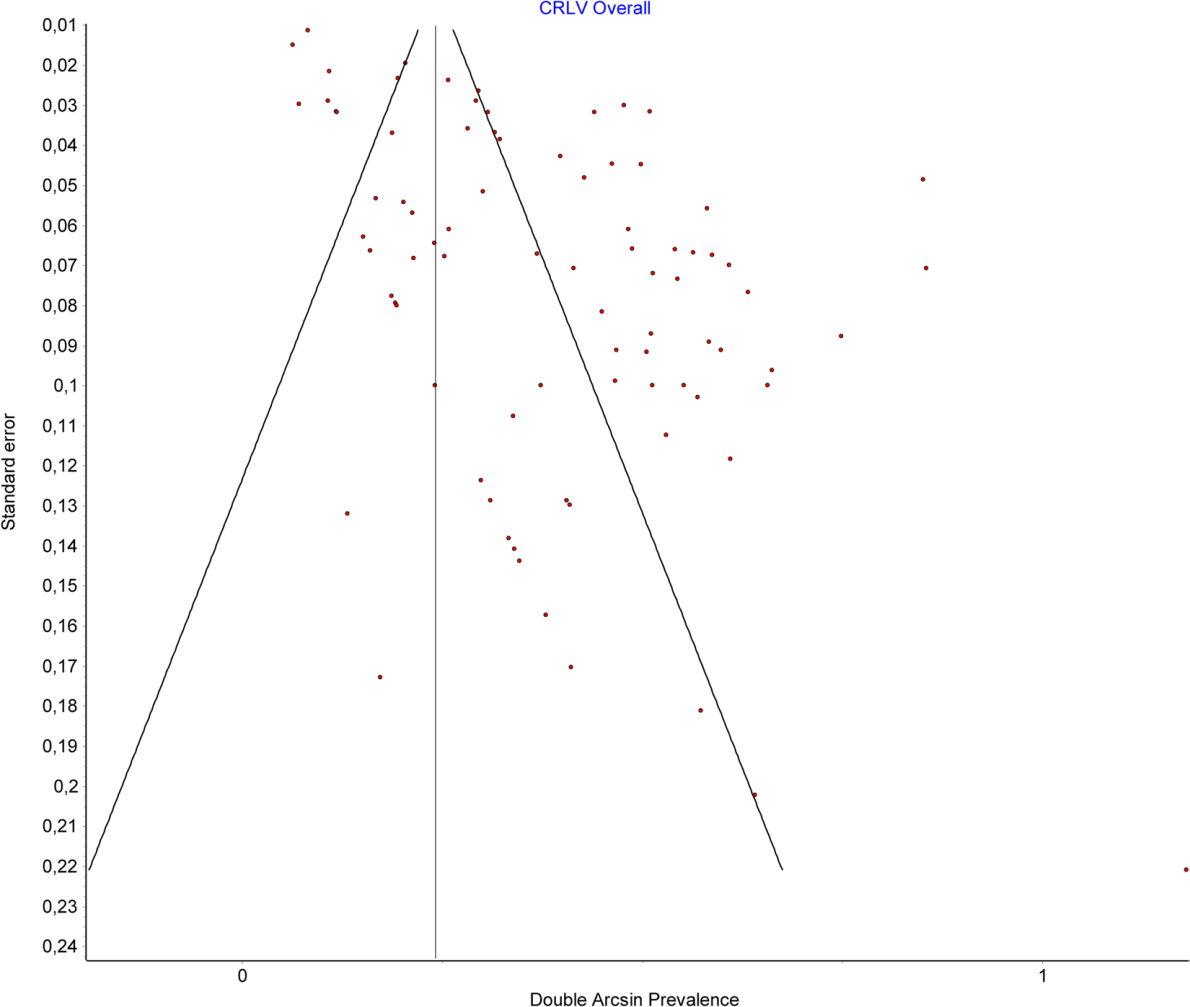
CLRV. Funnel plot.

A total number of 84 studies allowed us to estimate the prevalence of CLRV, containing 46256 subjects, of which 980 were positive. The overall prevalence for CLRV was 0.035 (CI:0.028–0.044) (Fig. 4). The publication bias was important, with a high number of studies being to the right of the funnel (Fig. 5), and having an LFK index of 4.24, suggesting major asymmetry. By comparing the prevalence depending on the method, we found for the autopsy group, a prevalence twice as high compared to CT and surgery, namely a prevalence of 0.05 (0.035–0.066) for autopsy, 0.026 (0.018–0.035) for CT, and 0.021 (0.005–0.040) for surgery. Fourteen studies separated the cases based on gender. For men, the overall prevalence was 0.036 (0.024–0.049), while for women −0.027 (0.014–0.042).

### Multiple renal veins

**Figure 6:**
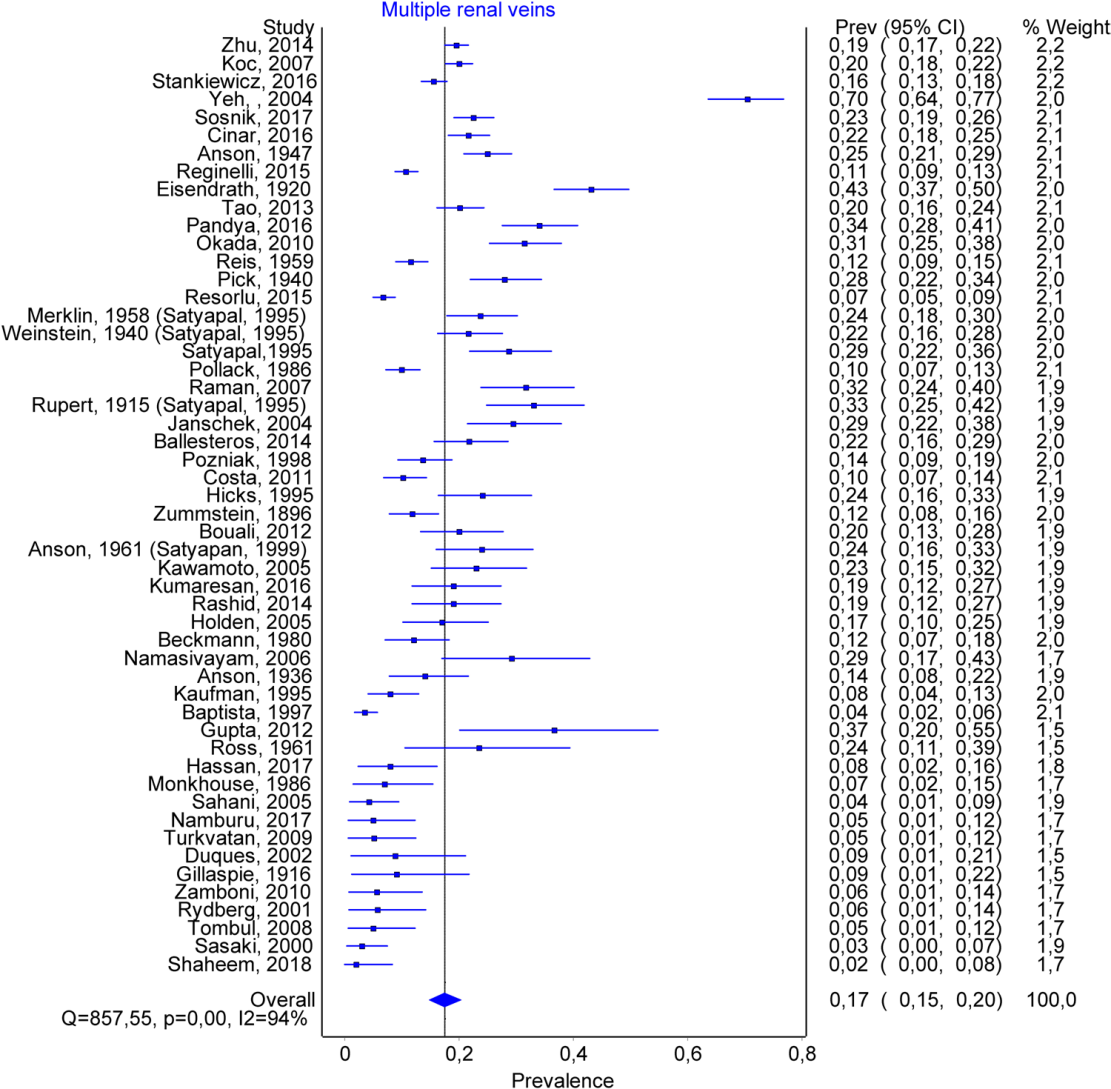
MRVs. Overall prevalence.

**Figure 7:**
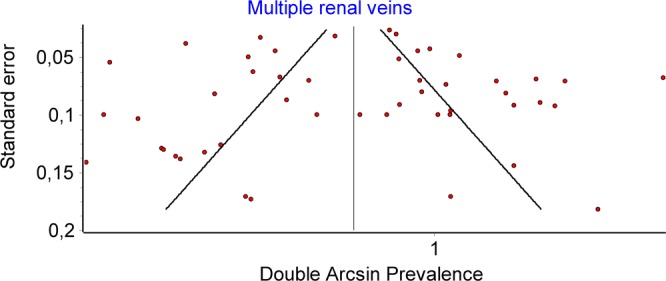
MRVs. Funnel plot.

**Figure 8:**
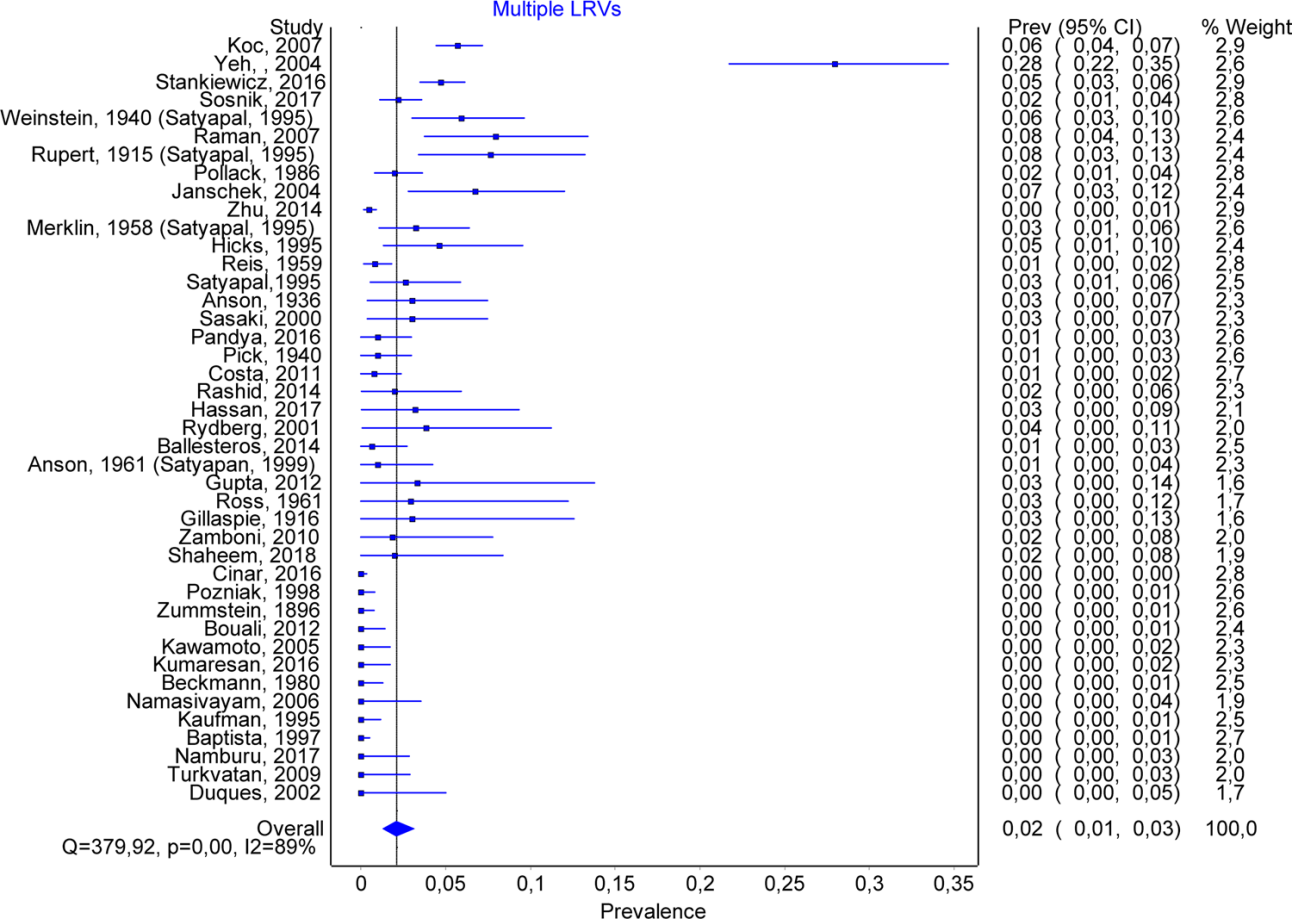
Multiple LRVs. Overall prevalence.

**Figure 9:**
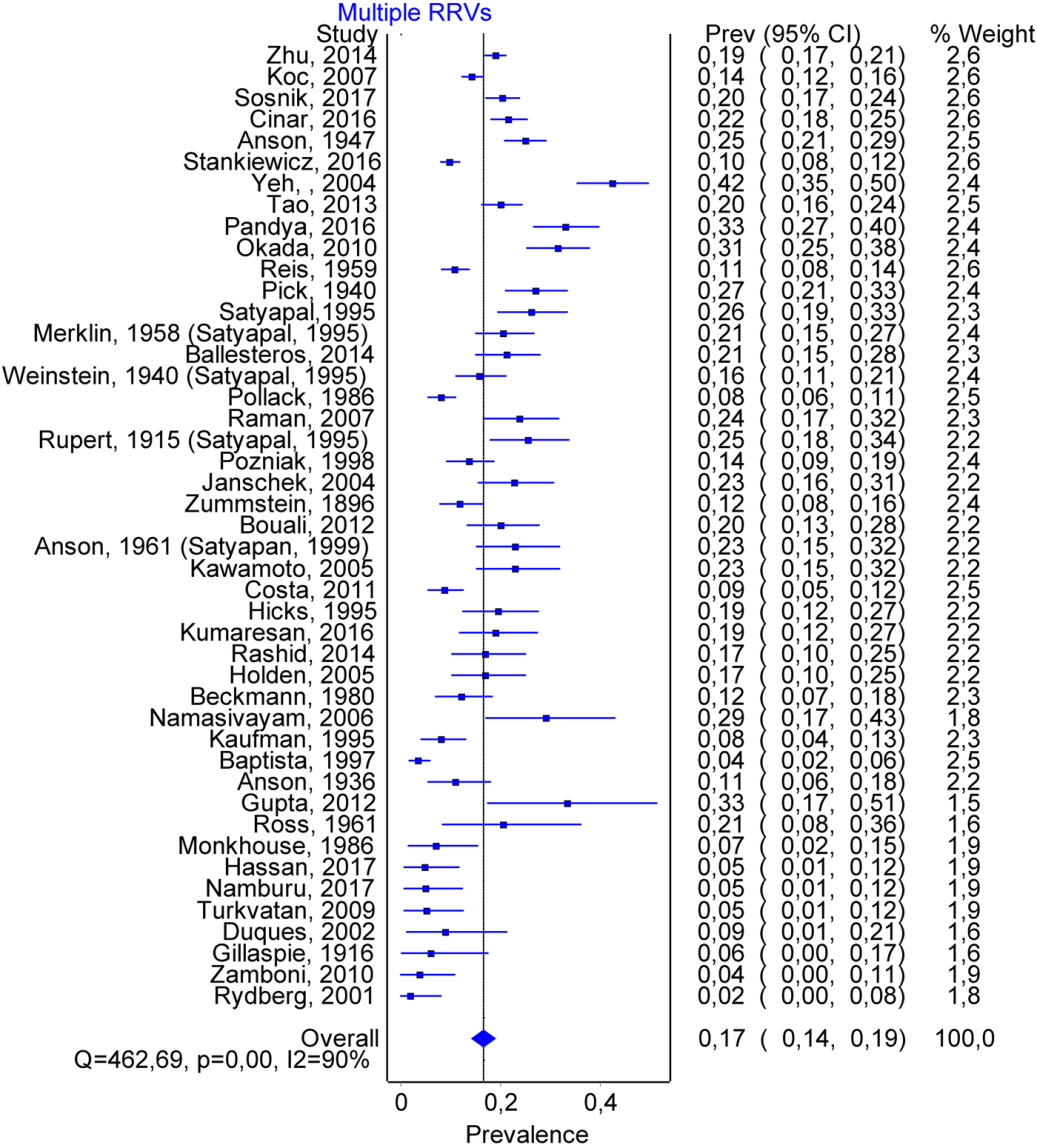
Multiple RRVs. Overall prevalence.

**Figure 10:**
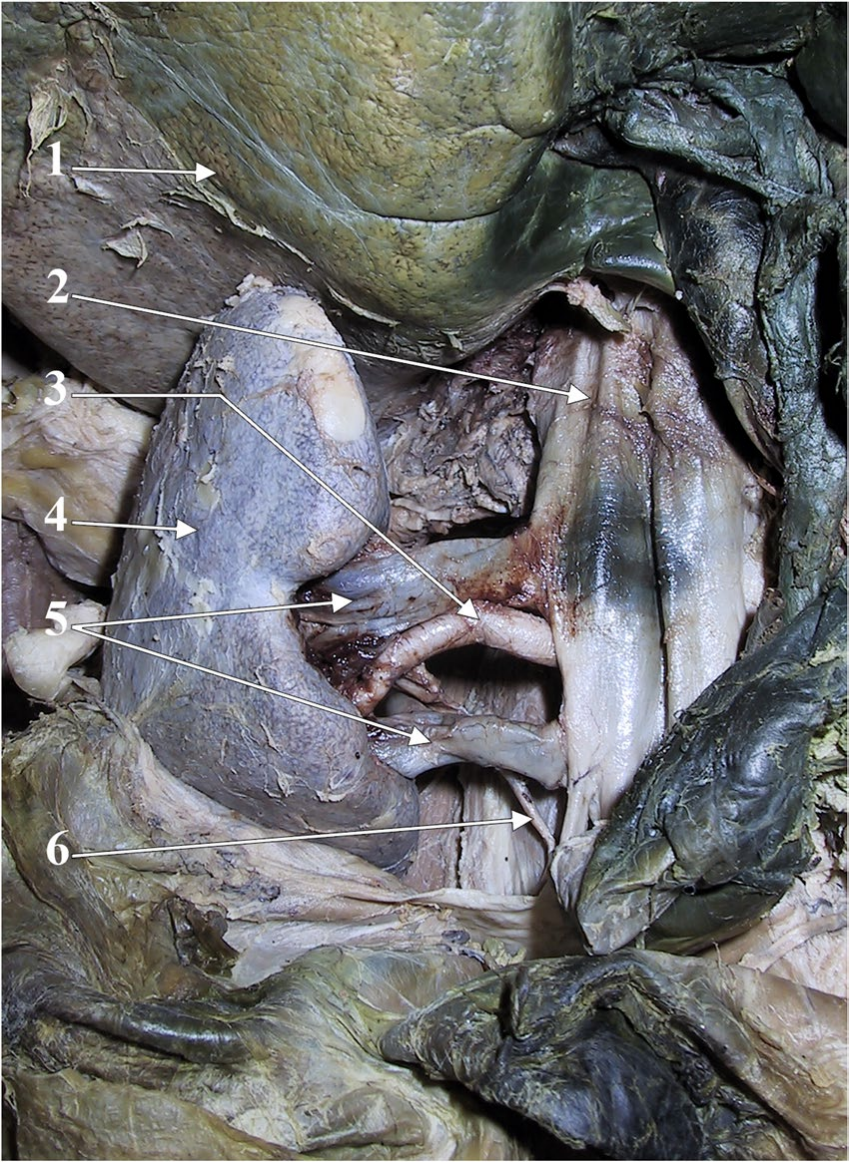
Dissection of the right renal vessels, anterior view. 1. liver; 2. inferior vena cava; 3. right renal a.; 4. right kidney; 5. double right renal vein; 6. right ureter (from the personal collection of MCR).

A total number of 51 articles allowed us to estimate the prevalence of double renal veins, containing 12773 subjects. Multiple renal veins were identified in 2241 cases, of which 1762 on the right side (RRV) and 221 on the left side (LRV). Double renal veins were encountered in 1450 cases (1317 on the right and 133 on the left side), and triple renal veins in 247 (170 on the right and 77 on the left). The overall prevalence of multiple renal veins was 0.167 (0.143–0.192) (Fig. 6). The publication bias was minor (Fig. 7), and an LFK Index of −1.04, suggesting minor asymmetry. Forty-two studies had data about multiple left renal veins. The overall prevalence was 0.021 (0.013–0.032) (Fig. 8), and publication bias was absent (LFK Index = 0.67, suggesting no asymmetry). Forty-four studies had data about multiple right renal veins. The overall prevalence was 0.166 (0.142–0.191) (Figs 9 and 10), and publication bias was −0.26, suggesting no asymmetry. The prevalence of double and triple renal veins is presented in Table 3.

**Table 3:** Prevalence of double and triples renal veins.

Variant	No Studies	Prevalence	LFK Index
Double LRV	35	0.017 (0.011–0.250)	0.67 (no asymmetry)
Triple LRV	33	0.004 (0.001–0.008)	1.53 (minor asymmetry)
Double RRV	38	0.138 (0.118–0.160)	0.90 (no asymmetry)
Triple RRV	35	0.017 (0.011–0.024)	−1.21 (minor asymmetry)

## Discussions

Our study is the first one to properly assess, using a statistical method, the prevalence of three main anatomical variants of the renal veins, namely RLRV, CLRV and MRVs.

These anatomical variants are important for surgeons, as their presence can alter the surgery protocol, and for clinicians, as they might lead to unforeseen clinical manifestations of various disorders (see Table 4 for details). In kidney donation, the morphology and size of the renal vessels is extremely important, as short vessels could increase the difficulty of vascular anastomosis and increase the warm ischemia time during the intervention^17^.

**Table 4:** Main clinical consequences of renal vein variants.

Variant	Clinical and surgery-related consequences
CLRV	• Risk of injury during surgery^31,110^. Sometimes, the posterior limb is not acknowledged before surgery and the renal vein seems to be normally looking, case in which the surgeon might injure the posterior limb^27^.
	• Changes needed of the standard surgery protocol for renal transplantation, aneurysm resection^31^
	• See also RLRV
RLRV	• It may have a high number of lumbar retroperitoneal tributaries, forming complex retroaortic systems, which can be easily injured during surgical dissection^27^
	• Changes needed of the standard surgery protocol for renal transplantation, aneurysm resection^31^
	• May have a sharp descending trajectory, joining the left common iliac vein, altering the surgical protocol^28^
	• The presence of a RLRV or CLRV is associated with a decrease of the infrarenal segment of the IVC, which could be an important consideration when placing a IVC filter, some of them being too long for the short infrarenal IVC segment^30^
	• RLRV has been associated with renal ectopy. For example, Macchi described a case of RLRV that was draining toward the IVS through two vessels which diverged into an acute angle and emptied independently into the IVC, forming a retroaortic juxtacaval aortic ring^5^.
	• Can be a correctable cause for varicocele. Arslan found a significant association between varicocele and RLRV^2^
	• Pelvic congestion syndrome (dysmenorrhea, lower abdominal pain, varices – vulvar, gluteal, thigh)^3^
	• Left gonadal reflux in men (lower limb varices, varicocele)^3^
	• Can mimic a cancer^34^
	• Fistula between the aorta and RLRV has been reported^111,112^
	• Can cause Nutcracker syndrome/phenomenon. There are two main forms of the Nutracker phenomenon: anterior NP, in which the LRV is caught in the fork between the abdominal aorta and the superior mesenteric artery, and posterior NP, in which there is a decreased space between the aorta and the spine, compressing the RLRV^113^; this leads to hematuria due to increased pressure in the LRV, causing congestion of the left kidney and the presence of venous communications^9^.
	• Can lead to renal vein hypertension^72^ with hematuria. For example, Gibo and Onitsuka described the case of a 13 years old girl who accused macrohematuria and low back pain; during the clinical investigation, it was found to have a RLRV, with compression of the vein between the aorta and the spine, causing an increased pressure gradient between the LRV and the IVC (mean of 6.8 mm Hg)^4^ (a value above 3 being indicative for renal vein hypertension).
	• Can lead to hematuria. For example, Karaman showed that compression of the RLRV is significantly associated with hematuria (out of 16 patients with compression of the RLRV, 15 patients were in the urological group); moreover, the urologic symptomatology was more frequent in RLRV types II and IV^9^.
	• Can cause left flank^52^ or low-back pain^4^
	• Can cause ureteropelvic junction obstruction^52^
	• Renin sampling from the renal vein^3^; a false lower renin level can be obtained with the catheter tip in the proximal portion of the left renal vein, due to additional supply from the left gonadal, second lumbar and hemiazygous veins^114^
	• Increase the intrarenal venous impedance index^103^, potentially causing nephrolithiasis or renal cysts^39^
MRVs	• Injury during surgery^31^
	• Changes needed of the standard surgery protocol for renal transplantation, aneurysm resection^31^

RRV is usually located anterior or inferior from the right renal artery^18^. RRV has less often an extrahilar origin (77.9%), compared to LRV (82.7%)^18^. It has an average length of 3.2cm^19^. Various studies showed RRV to be more often multiple, compared to the LRV; the main reason postulated for the increased prevalence of double RRV compared to LRV is the complex embryogenesis on the left side, discouraging the retention of additional left-sided renal veins^20^. Our study showed an overall prevalence of 16.7% for multiple renal veins, which were about eight times more frequent on the right compared to the left side. In kidney donors, the left one is preferable to be donated, due to a longer vascular pedicle. However, if the left kidney has a more complex vascular anatomy, the right one should be harvested. If the donor has one or both kidneys abnormal, the most normal remains to the donor, and the more abnormal one is given to the recipient^21^. Before donation, a complete imaging characterization of the kidneys and the vasculature should be performed, ideally through CT angiography, which yields data about the anatomy and variations of the renal vessels^21^. Some authors consider double right renal veins are a contraindication for donor nephrectomy, due to a higher risk of graft renal vein thrombosis^22^.

LRV can have either an intra or an extrarenal origin, with two or three main tributaries^17,23^, and is located anterior, or inferior of the renal artery, or it may run obliquely towards the IVC^18,24^. It has an average length of 8.4cm^19^, being much larger compared to the RRV, due to the abdominal topography of the IVC. The scientific literature has shown LRV to be less often double, but to present other variants, such as CLRV or RLRV.

According to Gillot, there are three main types of CLRV: (1) CLRV with partial distal bifidity, in which the retroaortic branch receives the root of the hemiazygos; (2) CLRV with partial proximal bifidity, a more common variant, in which the origin is separated, and the two branches join together in front of the aorta; (3) complete CLRV, in which we have two thick venous trunks that are leaving the hilum, and they remain separated until their ending in the IVC. This type has two subtypes: (a) inferior polar, in which the main vein, the superior one is preaortic, and the inferior polar vein is retroaortic; (b) superior polar, in which the main trunk is horizontal, preaortic; it receives the adrenal and sometimes the gonadal gland. The superior polar vein is retroaortic, and usually has an oblique, inferior course toward the IVC^25^. The actual prevalence of the CLRV depends on the attention with which the LRV is analyzed; if we were to consider all small retroaortic vessels draining into the IVC or LRV, the prevalence can be as high as 16%^26,27^. Other authors only included in the CLRV large, persistent collars, importantly decreasing the overall prevalence^27^. Our study confirmed a high variability regarding the reported prevalence of the CLRV and showed its actual prevalence to be around 3.5%.

RLRV can be classified in: RLRV Type 1, caused by the persistence of the left subsupracardinal anastomosis, the intersupracardinal anastomosis and the left dorsal renal vein, associated with the obliteration of the ventral left renal vein, leading to a retroaortic, orthotopic course for the LRV; RLRV Type 2, caused by the persistence of the subsupracardinal anastomosis on the left side, and of the left supracardinal vein, associated with the obliteration of the intersubcardinal and intersupracardinal anastomoses, leading to the appearance of a single retroaortic left renal vein lying at the L4-L5 level, where it joins the gonadal and ascending lumbar veins^10^; RLRV type III (CLRV); RLRV Type 4, in which the RLRV joins the left common iliac vein^9^, due to an obliteration of the ventral preaortic limb of the left renal vein^28^. The number of studies separating RLRVs into subtypes was small (five); additionally, some authors only scrutinized the first two subtypes, while other analyzed all four subtypes, and therefore we could not do a proper analysis of the prevalence on subtypes of RLRV.

Besides MRVs, RLRV, and CLRV, some authors described other variants, such as the presence of a plexiform left renal vein, with division after emerging from the renal hilum, followed by a redivision and a distal unification in a single terminal renal vein^29^.

The clinical consequences of renal vein abnormalities have been intensely studied; however, for many of them the scientific proofs are not definite. Their presence is however extremely important in the surgery of the abdomen, where they can be associated with significant complications, or the need to change the surgical approach. The main implications of these abnormalities are presented in Table 4.

The most important factor causing heterogeneity of the results regarding the prevalence of these variants is, most likely, represented by a variable number of false negative results, the variants being more easily overlooked when not specifically searched for.

### Limitations

Some studies did not specified number of cases, but rather a prevalence in percentage^17^; our reconstruction of primary data was done strictly arithmetical, by multiplying the total number of subjects with the percentage/100, with rounding to the superior value in the obtained number was above 0.5 and to the inferior value if the obtained number was below 0.5. The definition of various anatomical variants, and their classification, differed from study to study, and often there was no detailed description of the variant; therefore, our interpretation might not be exact (e.g. some studies included CLRV in the RLRV category), some studies included all CLRVs in their analysis while other included only those CLRV with both trunks of increased size, etc. Small retroaortic renal veins can be obscured due to volume averaging or limited resolution of the imaging techniques^30^. Many included studies were not designed specifically for the detection of caval abnormalities; many were retrospective, and included patients that were referred for abdominal or pelvic symptoms/disorders.

## Conclusions

The overall prevalence for RLRV is 3%, for CLRV −3.5%, and for MRVs −16.7%, much higher for the right (16.6%), compared to the left renal vein (2.1%).
